# Suppressive function of bone marrow-derived mesenchymal stem cell-derived exosomal microRNA-187 in prostate cancer

**DOI:** 10.1080/15384047.2022.2123675

**Published:** 2022-10-16

**Authors:** Chuangui Li, Zhen Sun, Yajun Song, Yong Zhang

**Affiliations:** a Department of Urology, Hebei Medical University, Shijiazhuang, P. R. China; b Department of Urology, Songshan General Hospital, Chongqing, P. R. China; c Department of Urology, the Second Affiliated Hospital, Army Medical University, Chongqing, P. R. China; d Department of Urology, National Cancer Center/National Clinical Research Center for Cancer/Cancer Hospital, Chinese Academy of Medical Sciences and Peking Union Medical College, Beijing, P. R. China

**Keywords:** Prostate cancer, human bone marrow-derived mesenchymal stem cells, exosomes, microRNA-187, CD276, JAK3-STAT3-Slug signaling pathway

## Abstract

Application of bone marrow-derived mesenchymal stem cell-derived exosomes (BMSC-exos) in cancer treatment has been widely studied. Here, we elaborated the function of BMSC-exos containing microRNA-187 (miR-187) in prostate cancer. Differentially expressed miRs and genes were screened with microarray analysis. The relationship between CD276 and miR-187 in prostate cancer was evaluated. Following miR-187 mimic/inhibitor or CD276 overexpression transfection, their actions in prostate cancer cell biological processes were analyzed. Prostate cancer cells were then exposed to BMSC-exos that were treated with either miR-187 mimic/inhibitor or CD276 overexpression for pinpointing the in vitro and in vivo effects of exosomal miR-187. miR-187 was poorly expressed while CD276 was significantly upregulated in prostate cancer. Additionally, restoring miR-187 inhibited the prostate cancer cell malignant properties by targeting CD276. Upregulation of miR-187 led to declines in CD276 expression and the JAK3-STAT3-Slug signaling pathway. Next, BMSC-exos carrying miR-187 contributed to repressed cell malignant features as well as limited tumorigenicity and tumor metastasis. Collectively, this study demonstrated that BMSC-derived exosomal miR-187 restrained prostate cancer by reducing CD276/JAK3-STAT3-Slug axis.

## Introduction

Prostate cancer is the second most prevalent cancer in men, and nearly all men diagnosed with prostate cancer are after the age of 50 years old, whose incidence and mortality are gradually elevated in most native Asian populations.^1^ Most prostate cancer patients can develop metastasis and die from the metastasis.^2^ Cell-based therapies can be used for the treatment of prostate cancer, especially mesenchymal stem cells (MSCs) due to their self-renewal potentials and transdifferentiation capacity.^3^ Specifically, human bone marrow-derived MSCs (hBMSCs) demonstrate therapeutic potential in prostate cancer.^4,5^

The therapeutic potential of MSCs has been identified to be related to their release of exosomes.^6,7^ Exosomes can transfer proteins, messenger RNAs (mRNAs), and microRNAs (miRs).^8,9^ As short, non-coding oligonucleotides, miRs are often dysregulated in many cancers including prostate cancer.^10^ Interestingly, miR-187-3p has been found to be enriched in MSC-exos.^11^ The expression of miR-187 is diminished in metastatic prostate cancer tissues and it can work as a recognizer of bone metastasis, serving as a key diagnostic marker of metastatic prostate cancer.^12,13^ CD276 has been pinpointed to be a putative target of miR-187 which can decrease mRNA expression of CD276 in ccRCC.^14^ CD276, also B7-H3, is a newly discovered molecule of B7 family and abundantly expressed in many different tumor tissues, including prostate cancer, suggestive of its potentials for cancer treatment.^15,16^ Downregulation of CD276 is capable of repressing the malignant phenotypes of prostate cancer cells.^17^ In addition, CD276 has been involved in the activation of JAK2-STAT3 pathway in multiple myeloma.^18^ Meanwhile, CD276 is able to facilitate the metastasis and invasion of HCC cells by activating the JAK3-STAT3-Slug pathway.^19^ Accumulating studies have highlighted the tumor-suppressive role of restraining the JAK3-STAT3 signaling pathway and the STAT3-Slug signaling pathway.^20–23^ However, the potential role of the JAK3-STAT3-Slug pathway in prostate cancer remains elusive. Based on the above findings, this study hypothesizes that hBMSC-derived exosomal miR-187 may eliminate the growth and metastasis of prostate cancer *via* modulation of the CD276-dependent JAK3-STAT3-Slug pathway. To address this hypothesis, the present study aimed to explore the exosomal communication within the prostate cancer microenvironment using hBMSCs, as well as the specific mechanism of hBMSC-derived exosomal miR-187 in prostate cancer cell growth and metastasis *via* the CD276/JAK3-STAT3-Slug axis.

## Materials and Methods

### Ethics Statement

All experiments were implemented with the ratification of the Ethics Committee of the Cancer Hospital, Chinese Academy of Medical Sciences and strictly in accordance with the *Declaration of Helsinki*. All patients provided written informed consent. Animal experiments conformed to the principles of animal protection and use, and were ratified by the laboratory animal Ethics Committee.

### In Silico Analysis

Gene Expression Omnibus database was used for the retrieval of prostate cancer-related microarray datasets, where miR microarray datasets (GSE45604 and GSE76260), as well as gene microarray dataset GSE30994 were employed for the differential difference analysis. The background correction and standardization of the pre-treatment microarray datasets were conducted using the R “Affy” package while the differentially expressed genes were screened using the R “limma” package with |logFoldChange (FC)| > 1 and *adj.P.Val* < 0.05 as screening criteria. Subsequently, the heat map of differential expression was constructed. The targeting relationship between differentially expressed miRs and target genes was computationally predicted using RNA22, miRSearch, miRDB, TarBase and DIANA databases. Lastly, starBase Pan-Cancer Analysis Platform was applied to discern the expression of differentially expressed miRs and genes in prostate cancer.

### Clinical Sample Collection

The prostate cancer tissue samples and the corresponding adjacent normal tissues (2 cm away from the tumor margin) were harvested from 57 patients (aged 50–84 years old) pathologically diagnosed with prostate cancer who had previously undergone surgical resection at Cancer Hospital, Chinese Academy of Medical Sciences. Among all patients, 28 cases were poorly differentiated, 18 were moderately differentiated and 11 were highly differentiated. Meanwhile, according to the tumor-node-metastasis staging, there were 16 cases in T1 stage, 21 cases in T2 stage, 12 cases in T3 stage and 8 cases in T4 stage. All included patients did not undergo chemotherapy or radiotherapy prior to the surgery. All tissue specimens were immediately frozen in liquid nitrogen for 1 h after saline washing and placed in a refrigerator at −80°C for long-term preservation.

### Screening of Cell Lines

Four prostate cancer cell lines (22Rv1, LNCaP, Du145, and PC-3) and normal prostatic epithelial cell line RWPE-1 were procured from Shanghai Institutes for Biological Sciences (Shanghai, China). The 22Rv1, LNCaP, Du145 and PC-3 cells were cultured in RPMI 1640 medium (Gibco, Carlsbad, California) while RPWE-1 cells in Keratinocyte serum-free medium (Gibco). Both media contained 10%FBS, 100 ug/mL streptomycine (Gibco) and 100 U/mL penicillin (Gibco). All cells were cultured in a 5% CO_2_ incubator at 37°C, with 95% saturated humidity. The media were renewed 3–4 times a week.

### Cell Transfection

Plasmids were transduced when logarithmically growing prostate cancer cells reached about 80% confluence. The prostate cancer cells were transduced with negative control (NC)-mimic, miR-187-mimic, NC-inhibitor, miR-187-inhibitor, overexpression (oe)-NC, oe-CD276, silenced (si)-NC, si-CD276, miR-187-mimic + oe-CD276 and NC-mimic + oe-CD276 employing a Lipofectamine 2000 Kit (11668–019, Invitrogen, New York, California). All the sequences and plasmids were procured from GenePharma Co. Ltd. (Shanghai, China).

### Luciferase Assay

The putative miR-187 binding sites to CD276 were predicted using the TargetScan database. The pGLO was purchased from Bio-Rad Company (Hercules, CA). Synthetic pGLO-CD276 3’-UTR wild-type (WT) and pGLO-CD276 3’-UTR mutant-type (MUT) were co-transfected with miR-187-mimic and NC-mimic into HEK-293 T cells (cell bank, Chinese Academy of Sciences) for 24 h. On the basis of Dual-Luciferase® Reporter Assay System (Promega Corporation, Madison, WI), luciferase activity was quantified.

### Cell Proliferation Assay

The statistical results of the PC-3 cell proliferation test were obtained by CCK-8 (CK04, Dojindo Laboratories, Kummoto, Japan). The optical density at 450 nm was assessed with the help of the microplate reader.

### Apoptosis Measurement of Flow Cytometry

Apoptosis of PC-3 cells was detected employing a Annexin V-FITC/PI detection kit (MA0220, Dalian Meilun Biotech Co., Ltd., Dalian, China).^24^

### Transwell Assay

Transwell invasion assay was processed with Transwell plate (Corning Glass Works, Corning, N.Y.) pre-coated with Matrigel (YB356234, YuBo Biological Technology Co., Ltd., Shanghai, China) for assessing cell invasion capacity.^25^ Analysis of the results was started in at least 4 randomly selected visual fields under an inverted microscope.

### Wound-healing Assay

PC-3 cells were seeded into a 6-well plate (2.5 × 10^4^ cells/mL) and incubated for 24 h. Scratches were made using 10 mL of sterilized disposable pipettes. The cells were further incubated with complete medium. Each well at 0 and 24 h after the wound were imaged under an inverted microscope. The width of each scratch was analyzed utilizing ImageJ software.

### Isolation and Identification of hBMSCs

In this experiment, the hBMSCs were isolated from bone marrow aspirates donated by healthy volunteers aged 29 to 83 years from Cancer Hospital, Chinese Academy of Medical Sciences. Aspiration was performed using a 15 gauge needle, and after aspiration of every 2 mL bone marrow, the needle was carefully rotated to minimize contamination by peripheral blood. The volunteers were excluded if they met the following criteria: individuals used bisphosphonates, estrogens (oral or transdermal), raloxifene or parathyroid hormone (PTH) (or other bone active drugs) in the past three years; 2) individuals had a history of Paget’s Disease, other metabolic bone diseases, diabetes or major heart, kidney or liver diseases; 3) individuals had a history of any fracture in the past five years; 4) individuals received hysterectomy; 5) individuals had abnormalities in the screening laboratory studies.^26^

The obtained hBMSCs were incubated on tissue-treated culture plates containing RPMI-1640 replenished with penicillin/streptomycin and 10% FBS. The plates were cultured (48 h) at 37°C with humidity and 5% CO_2_. The non-adhered cells were then removed, followed by replacement of the medium. Upon 80–90% confluence, the hBMSCs were detached by exposure to 0.05% trypsin for subculture. Thereafter, hBMSCs with no more than five passages were used in the remaining experiments. Observation of cell morphology was implemented under a light microscopy (Olympus Corporation, Tokyo, Japan).^27^

The immune profile of hBMSCs was detected using flow cytometry. In brief, after detachment, hBMSCs were washed with PBS without calcium and magnesium, followed by blocking with 10% normal goat serum (Abcam Inc., Cambridge, UK) to restrain the nonspecific binding. The hBMSCs were then incubated with FITC-tagged monoclonal antibodies against CD29, CD44, CD90, CD34, and CD45 (1: 100, BioLegend, San Diego, CA) for 30 min, followed by analysis on CyAn ADP Analyzer (Beckman Coulter, Brea, CA).^27^

### Extraction and Characterization of hBMSC-derived Exosomes (hBMSC-exos)

Exosomes were extracted from hBMSCs upon centrifugation, as previously described. Immunoblotting was utilized to validate the expression of exosome-specific surface markers (TSG101, CD63 and CD9) in 50 µg exosomes for the identification of exosome characteristics. A Zetasizer Nano ZS (Malvern Instruments, Malvern, UK) was utilized to measure the particle size of exosomes. Under a transmission electron microscope (TEM, Tecnai Spirit, FEI, Hillsboro, OR), the exosome morphology was observed.^28,29^

### Assessment of hBMSCs-exo Uptake by Prostate Cancer Cells

PKH67 dye (BB-441112, Bestbio, Shanghai, China) was mixed with suspension of exosomes. The mixture was placed at 2–8°C for 15–30 min, centrifuged to discard supernatant, and centrifuged at 100000 × g for 70 min. Co-culture of PKH67-traced exosomes with PC-3 cells was performed, and the uptake of exosomes by prostate cancer cells was monitored under a fluorescence microscope at 12, 24 and 48 h after co-culture.

### Co-culture of hBMSC-exos and Prostate Cancer Cells

When reaching about 60% confluence in a 24-well plate, PC-3 cells were co-cultured with the extracted exosomes for 48 h, including exosomes derived from NC-mimic-treated hBMSCs (hBMSC-exo-NC-mimic), exosomes derived from miR-187-mimic-treated hBMSCs (hBMSC-exo-miR-187-mimic), exosomes derived from NC-inhibitor-treated hBMSCs (hBMSC-exo-NC-inhibitor) and exosomes derived from miR-187-inhibitor-treated hBMSCs (hBMSC-exo-miR-187-inhibitor).

### Quantification of RNA Expression and Protein Expression

Total RNA extraction, cDNA synthesization and RT-qPCR were implemented as described previously.^30^ U6 was as applied for the normalizer of miR-205, while GAPDH of the remaining genes. With 2 μg of cDNA as a template, the relative transcriptional level of the target gene (Supplementary Table S1) was analyzed by 2^−ΔΔCt^ method.^31^

Total protein of cells and tissues was extracted. Extracts were resolved with SDS-PAGE and then electroblotted to a PVDF membrane which was incubated with the primary rabbit monoclonal antibodies (Abcam Inc., Cambridge, UK) to CD276 (ab134161, 1: 200), E-cadherin (ab40772, 1: 500), N-cadherin (ab18203, 1: 1000), Slug (ab27568, 1: 500), Ki67 (ab16667), Bcl-2 (ab182858, 1: 2000), Bax (ab32503, 1: 5000), JAK3 (ab45141, 1: 2000), STAT3 (ab68153, 1: 2000), p-STAT3 (ab76315, 1: 2000), VEGF (ab32152, 1: 1000), Fibronectin (ab24130), MMP-2 (ab92536, 0.5 µg/mL) and GAPDH (ab9485, 1: 2500) overnight at 4°C as well as with HRP-labeled goat anti-rabbit IgG antibody (ab205718, 1: 20000) at ambient temperature (1 h). The samples were developed with development solution (Roche Diagnostics GmbH, Mannheim, Germany). ImageJ 1.48 u Software (National Institutes of Health, Bethesda, Maryland) was employed for the quantitative analysis of protein.

### Immunohistochemistry

The tissue sections were immunostained with rabbit polyclonal antibodies (1: 50; ab28364) to CD31 and CD276 at 4°C overnight and then with goat anti-rabbit IgG antibody at 37°C (20 min), developed with DAB, and counterstained with hematoxylin, followed by a microscopic observation.^32^

### Animal Experiments

Thirty male BALB/c nude mice (6-week-old, 18–23 g) were randomized into 6 groups with 5 in each group. Single cell suspension (0.2 mL) with 1 × 10^6^ PC-3 cells was inoculated subcutaneously into nude mice to establish the subcutaneous transplanted tumor model. The hBMSC-exos were manipulated with miR-187 (Exo-miR-187 agomir), empty vector of miR-187 (Exo-NC-agomir), CD276 (oe-CD276), or empty vector of CD276 (oe-NC). The tail vein of nude mice was injected with normal saline, 30 μg Exo, 30 μg Exo-miR-187 agomir, 30 μg Exo-NC-agomir, 30 μg Exo-NC-agomir + oe-CD276, 30 μg Exo-NC-agomir + oe-NC on the 5th, 10^th^, 15^th^, 20^th^ and 25^th^ day respectively (n = 5).^33^ On the 28^th^ day, the mice were euthanatized. The tumors were isolated and weighed. Routine paraffin embedding and sectioning of tumor tissues were conducted for the histopathological analysis.

The *in vivo* tumor metastasis test was then carried out by injecting the stably transfected 2 × 10^6^ prostate cancer cells into the tail vein of male nude mice (n = 30), according to the above time point and dosage. The 30 male BALB/c nude mice at SPF grade were randomized into 6 groups (5 mice/pergroup). After 8 weeks, the mice were euthanatized, and the lungs of all mice were harvested for the evaluation of metastatic nodules.

### Statistical Analysis

Measurement data were depicted as mean ± standard deviation. Upon normal distribution and homogeneity of variance, paired or unpaired *t*-test, one-way analysis of variance (ANOVA) or repeated measures ANOVA or post-hoc test were processed. Wilcoxon rank-sum test was processed for data with skewed distribution and defect variances. Survival analysis was completed utilizing Kaplan-Meier method followed by Log-rank method for testing differences in survival rates. Counting data were analyzed by chi-square test. *p* < .05 described statistically significant.

## Results

### Low Expression of miR-187 in Prostate Cancer and Its Association with the Poor Prognosis of Patients with Prostate Cancer

**Figure 1.**
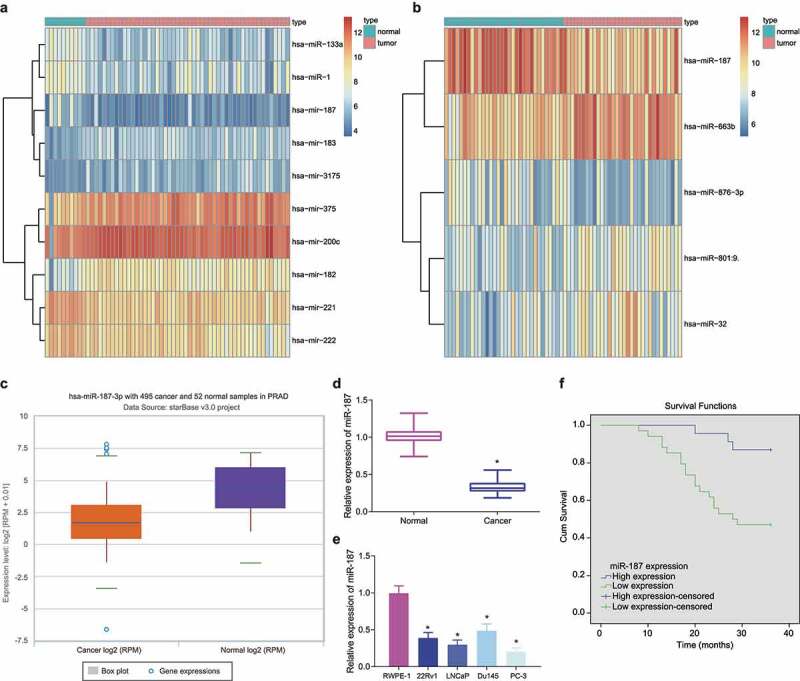
Low expression of miR-187 was observed in prostate cancer. A, A heat map of the expression of the differentially expressed miRs in the GSE45604 microarray dataset. B, A heat map of the expression of the differentially expressed miRs in the GSE76260 microarray dataset. The ordinate represents the gene name. The histogram on the upper right is color gradation, with each rectangle representing a corresponding sample expression value. C, miR-187 expression in prostate cancer analyzed by starBase Pan-Cancer Analysis Platform. D, miR-187 expression in prostate cancer and adjacent normal tissues measured by RT-qPCR (N = 57). E, miR-187 expression in prostate cancer cell lines (22Rv1, LNCaP, Du145, and PC-3) and normal prostatic epithelial cell line RWPE-1 measured by RT-qPCR. F, The correlation of miR-187 expression with the prognosis of patients with prostate cancer analyzed by Kaplan-Meier curve. Panel D-F was measurement data and expressed as mean ± standard deviation. Paired *t*-test was used for Panel D, and in panel E, one-way ANOVA was used for the comparison among multiple groups, followed by Turkey’s post-hoc test. In panel F, the log-rank method was used for survival difference test. * *p* < .05 *vs*. adjacent normal tissues or normal prostatic epithelial cell line RWPE-1. The experiments were repeated three times.

Differential analysis of GSE45604 and GSE76260 microarray datasets yielded 10 and 5 differentially expressed miRs, respectively (Figure 1a,). Both microarray datasets demonstrated reduced miR-187 expression in prostate cancer tissues. Additionally, such reduction in miR-187 expression was also confirmed by starBase Pan-Cancer Analysis Platform in prostate cancer (Figure 1c). Therefore, we chose miR-187 for subsequent experimentations.

Following RT-qPCR, lower miR-187 expression was detected in prostate cancer tissues (Figure 1d). Similar decrease in miR-187 expression was witnessed in 22Rv1, LNCaP, Du145, and PC-3 cells than in RWPE-1 cells, with the lowest miR-187 expression found in PC-3 cells and the highest one in Du145 cells (Figure 1e). Therefore, PC-3 and Du145 cells were chosen for the following experimentations.

Subsequently, based on the average expression of miR-187, 57 prostate cancer patients were separated into the high and low levels of miR-187 groups. The Kaplan-Meier curve demonstrated that poor prognosis of prostate cancer patients was accompanied by lower expression of miR-187 (figure 1f).

Hence, miR-187 was poorly expressed in prostate cancer and this poor expression indicated poor prognosis of prostate cancer patients.

### Overexpression of miR-187 Inhibits Prostate Cancer Cell Malignant Properties

**Figure 2.**
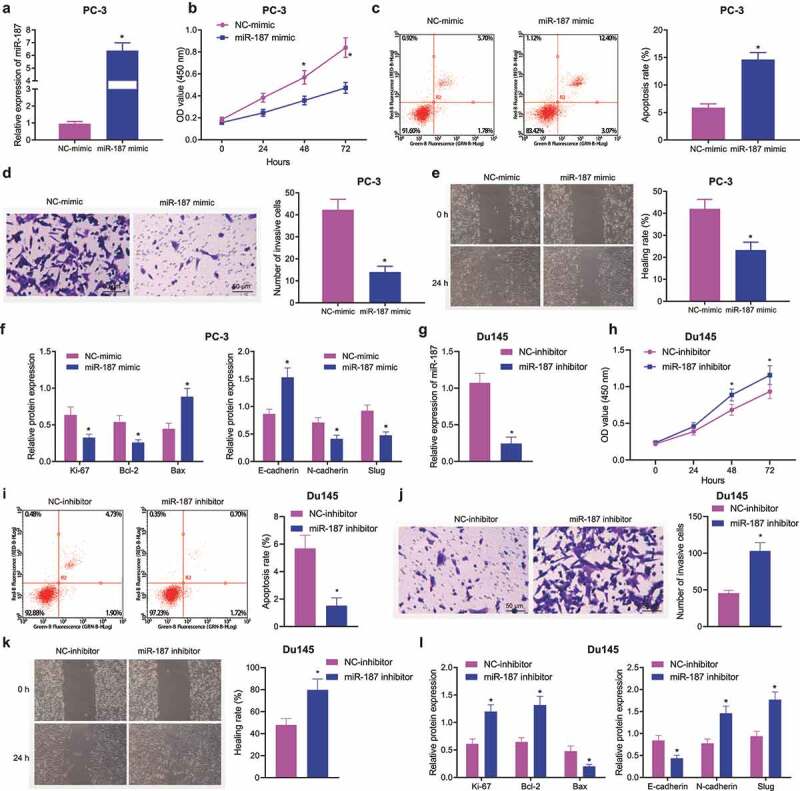
Overexpression of miR-187 contributes to suppression of prostate cancer cell proliferation, invasion and migration and promoted cell apoptosis. PC-3 cells were treated with NC-mimic or miR-187 mimic. A, miR-187 expression in PC-3 cells evaluated by RT-qPCR. B, Viability of PC-3 cells evaluated by CCK-8 assay. C, Apoptosis of PC-3 cells evaluated by flow cytometry. D, Invasion of PC-3 cells evaluated by Transwell assay (200×). E, Migration of PC-3 cells evaluated by Wound-healing assay. F, The protein expression of proliferation/apoptosis-related genes (Ki67, Bcl-2 and Bax) and EMT-related genes (E-cadherin, N-cadherin and Slug) in PC-3 cells evaluated by Western blot analysis. Du145 cells were treated with NC inhibitor or miR-187 inhibitor. G, miR-187 expression in Du145 cells evaluated by RT-qPCR. H, Viability of Du145 cells evaluated by CCK-8 assay. I, Apoptosis of Du145 cells evaluated by flow cytometry. J, Invasion of Du145 cells evaluated by Transwell assay (200×). K, Migration of Du145 cells evaluated by Wound-healing assay. L, The protein expression of proliferation/apoptosis-related genes (Ki67, Bcl-2 and Bax) and EMT-related genes (E-cadherin, N-cadherin and Slug) in Du145 cells evaluated by Western blot analysis. The measurement data were expressed as mean ± standard deviation. Comparison between two groups was analyzed using independent sample *t*-test. Comparison among groups at different time points was analyzed using two-way ANOVA. * *p* < .05 *vs*. NC mimic-treated PC-3 cells or NC inhibitor-treated Du145 cells. The experiments were repeated three times.

To confirm the aforementioned hypothesis, we overexpressed miR-187 in PC-3 cells or knocked down miR-187 in Du145 cells to discern the potentials of miR-187 in the biological characteristics of prostate cancer cells. As depicted in Figure 2a, RT-qPCR results suggested that miR-187-mimic was successfully transfected into PC-3 cells with high efficiency. Results in Figure 2b-e showed that mimic of miR-187 augmented PC-3 cell apoptosis and limited PC-3 cell viability, invasion, and migration. Meanwhile, immunoblotting data revealed that the protein expression of E-cadherin and Bax was upregulated but that of Ki67, Bcl-2, N-cadherin and Slug was diminished in the miR-187-mimic-treated PC-3 cells (figure 2f and Supplementary Figure S1A).

In addition, miR-187-inhibitor was successfully transfected in Du145 cells with high efficiency (Figure 2g). We found that miR-187-inhibitor increased Du145 cell viability (Figure 2h), migration (Figure 2j), and invasion (Figure 2k) but reduced apoptosis (Figure 2i). Meanwhile, in miR-187-inhibitor-treated Du145 cells, the levels of E-cadherin and Bax were downregulated but those of Ki67, Bcl-2, N-cadherin and Slug were upregulated (Figure 2l and Supplementary Figure S1B).

Collectively, overexpression of miR-187 inhibited the malignant phenotype of prostate cancer cells.

### CD276 was a Putative Target of miR-187 in Prostate Cancer Cells

**Figure 3.**
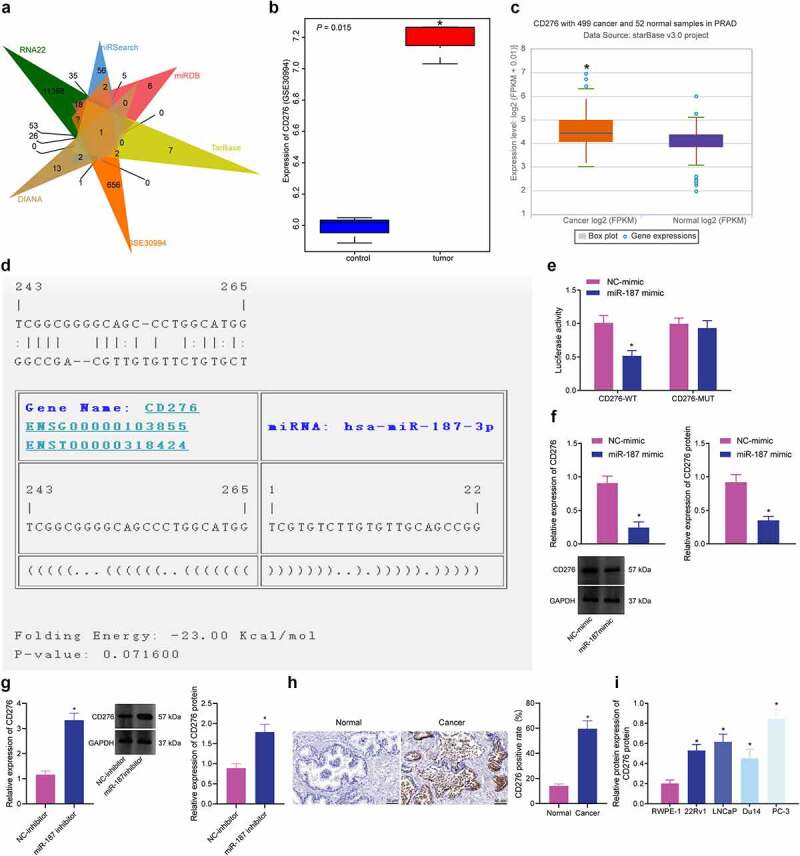
CD276 is a target gene of miR-187 in prostate cancer cells. A, Venn diagram of target genes of miR-187 predicted by the RNA22, miRSearch, miRDB, TarBase and DIANA databases and the differentially expressed genes in prostate cancer samples obtained from the GSE30994 microarray dataset. B, CD276 expression in prostate cancer samples in the GSE30994 microarray dataset. C, CD276 expression in prostate cancer samples analyzed by starBase Pan-Cancer Analysis Platform. D, The specific binding sites between CD276 and miR-187 predicted by the TargetScan database. E, The binding of miR-187 to CD276 verified by dual-luciferase reporter assay. F, The effect of miR-187 mimic on CD276 expression in PC-3 cells analyzed by RT-qPCR and Western blot analysis. G, The effect of miR-187 inhibitor on CD276 expression in Du145 cells was detected by RT-qPCR and Western blot analysis. H, CD276 expression in prostate cancer and adjacent normal tissues measured by immunohistochemistry (200×). I, The expression of CD276 in prostate cancer cell lines and normal prostate epithelial cell line assessed by Western blot analysis. The measurement data (E-G, I) were expressed as mean ± standard deviation. Comparison between two groups was analyzed using independent sample *t*-test, and one-way ANOVA was used for comparison among multiple groups, followed by Turkey’s post-hoc test. The count data (h) were analyzed by Chi-square test. * *p* < .05 *vs*. adjacent normal tissues, normal prostatic epithelial cell line RWPE-1, NC mimic-treated PC-3 cells or NC inhibitor-treated Du145 cells. The experiments were repeated three times.

To elucidate the possible mechanism of miR-187, the target genes of miR-187 were predicted by the RNA22, miRSearch, miRDB, TarBase and DIANA databases. After that, CD276 was found at the intersection of the predicted results with differentially expressed genes from the GSE30994 microarray dataset (Figure 3a). In addition, in the GSE30994 microarray dataset, CD276 was elevated in prostate cancer samples (Figure 3b), which was also verified by the starBase Pan-Cancer Analysis Platform (Figure 3c). Hence, we speculated that miR-187 might affect prostate cancer *via* regulation of CD276.

TargetScan database predicted specific binding sites between CD276 and miR-187 (Figure 3d). Luciferase assay unfolded that the luciferase activity of CD276-3’-UTR WT was inhibited following transfection with mimic of miR-187 (Figure 3e). As shown in figure 3f, CD276 expression was decreased in PC-3 cells following miR-187 overexpression. After treating Du145 cells with inhibitor of miR-187, the expression of CD276 was increased (Figure 3g), indicating that miR-187 targeted CD276 and reduced its expression. Immunohistochemistry results depicted that the positive rate of CD276 in prostate cancer tissues was 59.65%, while that in adjacent normal tissues was 14.04% (Figure 3h). Additionally, immunoblotting results depicted that CD276 was highly expressed in prostate cancer cell lines relative to RWPE-1 cells, and the highest expression was found in PC-3 cells (Figure 3i and Supplementary Figure S1C).

To sum up, CD276 was upregulated in prostate cancer and was targeted by miR-187.

### miR-187 Inhibits the JAK3-STAT3-Slug Signaling Pathway via Targeting CD276, Thus Impeding the Malignant Phenotypes of Prostate Cancer Cells

**Figure 4.**
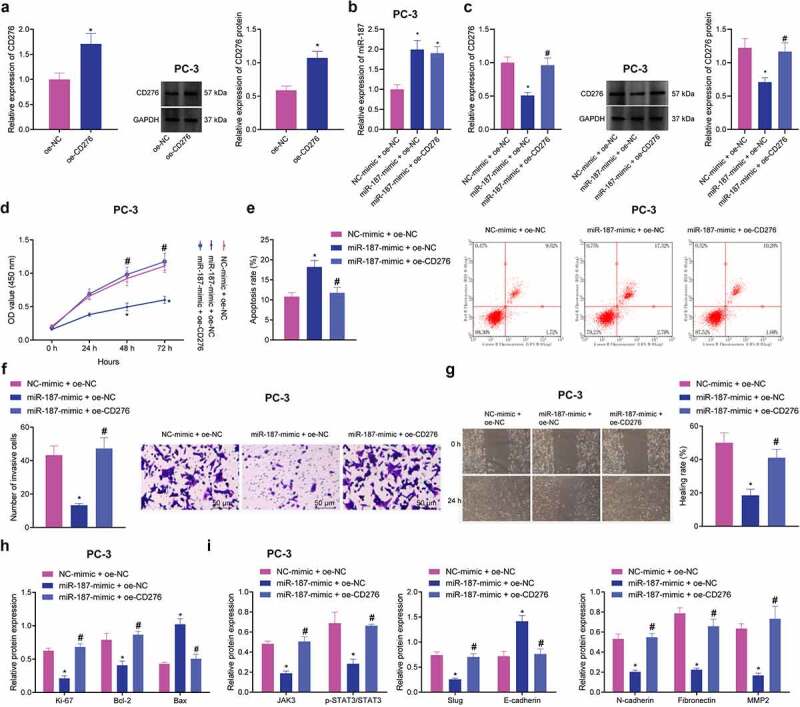
miR-187 downregulates CD276 and inactivates the JAK3-STAT3-Slug signaling pathway to suppress the malignant phenotype of prostate cancer cells. A, CD276 overexpression efficiency in PC-3 cells verified by RT-qPCR and Western blot analysis. PC-3 cells were treated with miR-187 mimic + oe-NC or miR-187 mimic + oe-CD276. B, miR-187 expression in PC-3 cells evaluated by RT-qPCR. C, CD276 expression in PC-3 cells evaluated by RT-qPCR and Western blot analysis. D, PC-3 cell viability evaluated by CCK-8 assay. E, PC-3 cell apoptosis evaluated by flow cytometry. F, PC-3 cell invasion evaluated by Transwell assay (200×). G, PC-3 cell migration evaluated by Wound-healing assay. H, The expression of apoptosis-related genes (Ki67, Bcl-2 and Bax) in PC-3 cells evaluated by Western blot analysis. I, The expression of the JAK3-STAT3-Slug signaling pathway-related genes in PC-3 cells evaluated by Western blot analysis. The measurement data were expressed as mean ± standard deviation. Cell viability at different time points among multiple groups was compared using two-way ANOVA, and comparison between two groups was analyzed using independent sample *t*-test. Comparison among multiple groups was analyzed using one-way ANOVA with Tukey’s post hoc test. * *p* < .05 *vs*. PC-3 cells treated with oe-NC or NC mimic + oe-NC. ^#^ *p* < .05 *vs*. PC-3 cells treated with miR-187 mimic + oe-NC. The experiments were repeated three times.

The aforementioned results confirmed that miR-187 targeted CD276 and downregulated its expression. Meanwhile, CD276 is overexpressed in many malignant tumors, and this overexpression accelerate the metastasis of cancer cells *in vitro*, which is often linked to poor clinical prognosis.^32,34^ These lines of evidence allowed us to discern the effect of miR-187 targeting CD276 on the biological function of prostate cancer cells. First, the overexpression efficiency of CD276 in PC-3 cells was verified by RT-qPCR and immunoblotting (Figure 4a). Additionally, we further found that treatment with miR-187-mimic + oe-NC in PC-3 cells increased miR-187 expression while decreasing CD276 expression; however, treatment with miR-187-mimic + oe-CD276 led to upregulated CD276 expression without altering miR-187 expression (Figure 4b,). Moreover, miR-187 overexpression in PC-3 cells repressed cell viability, invasion and migration while augmenting cell apoptosis but these results were reversed in the presence of further overexpression of CD276 (Figure 4d-g).

A previous study has documented that CD276 can accelerate the cell metastasis by activating the JAK-STAT signaling pathway in multiple myeloma cells.^35^ Immunoblotting revealed that after overexpression of miR-187 in PC-3 cells, the expression of Ki-67, Bcl-2, JAK3, p-STAT3, Slug, N-cadherin, Fibronectin, and MMP2 was reduced, while that of Bax and E-cadherin was elevated (Figure 4h-i and Supplementary Figure S1D-E). Conversely, the effect of overexpression of miR-187 was reversed by overexpression of CD276.

Collectively, miR-187 can decrease CD276 expression to inhibit the JAK3-STAT3-Slug signaling pathway, thus delaying the malignant phenotypes of prostate cancer cells.

### hBMSC-exos Deliver miR-187 into the Prostate Cancer Cells

**Figure 5.**
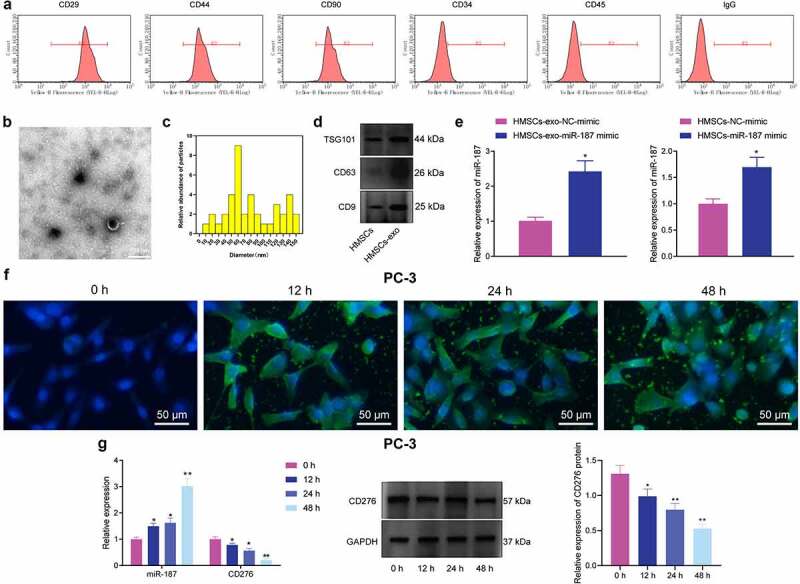
hBMSC-exos transfer miR-187 into prostate cancer cells. A, Expression of hBMSC surface markers CD29, CD44, CD90, CD34 and CD45 detected by flow cytometry. B, Ultrastructure of hBMSC-exos observed under a TEM (scale bar = 200 nm). C, Particle size of hBMSC-exos analyzed by Zetasizer Nano ZS. D, The expression of exosome-specific surface markers (TSG101, CD63 and CD9) in hBMSC-exos detected using Western blot analysis. E, miR-187 expression in hBMSCs and purified hBMSC-exos treated with miR-187 mimic detected by RT-qPCR. F, The uptake of hBMSC-exos by prostate cancer cells at 0, 12, 24 and 48 h observed by a fluorescence microscope (200×, PKH67: green fluorescent, DAPI: blue fluorescence). G, The expression of miR-187 and CD276 in PC-3 cells co-cultured with hBMSCs-exo-miR-187-mimic at 0, 12, 24 and 48 h detected by RT-qPCR and Western blot analysis. Measurement data were expressed as mean ± standard deviation. Comparisons between two groups were analyzed using independent sample *t*-test. Comparison among multiple groups was analyzed using one-way ANOVA with Tukey’s post hoc test. * *p* < .05 *vs*. PC-3 cells treated with hBMSCs-exo-NC-mimic or at 0 h. The experiments were repeated three times.

BMSCs have been reported to metastasize to the prostate cancer microenvironment, which may make them as gene carriers in gene therapy.^36^ A recent study identified BMSC-exos carried miRs to promote dormancy of breast cancer cells,^37^ suggesting that exosomal miR derived from hBMSCs could be used for the cancer treatment. We isolated BMSCs from human bone marrow and the subsequent characterization utilizing flow cytometry demonstrating that CD29 (100%), CD44 (98.9%), and CD90 (97.4%) were positively expressed, while CD34 (20.7%), and CD45 (19.5%) were negatively expressed (Figure 5a), suggestive of the successful isolation of BMSCs.

The exosomes were then isolated from the hBMSCs. Observation under the TEM demonstrated that the membranous vesicles with obvious heterogeneity in size and diameter ranging from 40 to 150 nm. These membranous vesicles presented round or elliptical in shape with similar morphology. The membranous structure was observed around the vesicle, with low-density substances in the center (Figure 5b). Subsequent analysis employing Zetasizer Nano ZS displayed that the size was about ~62.58 nm (Figure 5c). Furthermore, immunoblotting showed that the expression of TSG101, CD63, and CD9 was higher in hBMSC-exos than in hBMSCs (Figure 5d), confirming the successful isolation of exosomes.

Results noted that miR-187 expression was elevated in hBMSCs and hBMSC-exos treated with miR-187 mimic (Figure 5e). PC-3 cells were co-cultured with PKH67-labeled hBMSC-exos. Under a fluorescence microscopy, uptake of exosomes by PC-3 increased over time (figure 5f). Besides, exosomes were extracted from hBMSCs overexpressing miR-187 and co-cultured with PC-3 cells, and RT-qPCR and immunoblotting were implemented for quantifying the miR-187 and CD276 expression in PC-3 cells at 0, 12, 24 and 48 h. It was found that in PC-3 cells, the miR-187 expression was up-regulated while that of CD276 was diminished over time (Figure 5g).

Taken together, hBMSC-exos could transfer miR-187 into prostate cancer cells.

### miR-187 delivered by hBMSC-exos Inhibits Malignant Phenotypes of Prostate Cancer Cells by Limiting CD276-dependent Activation of the JAK3-STAT3-Slug Signaling Pathway

**Figure 6.**
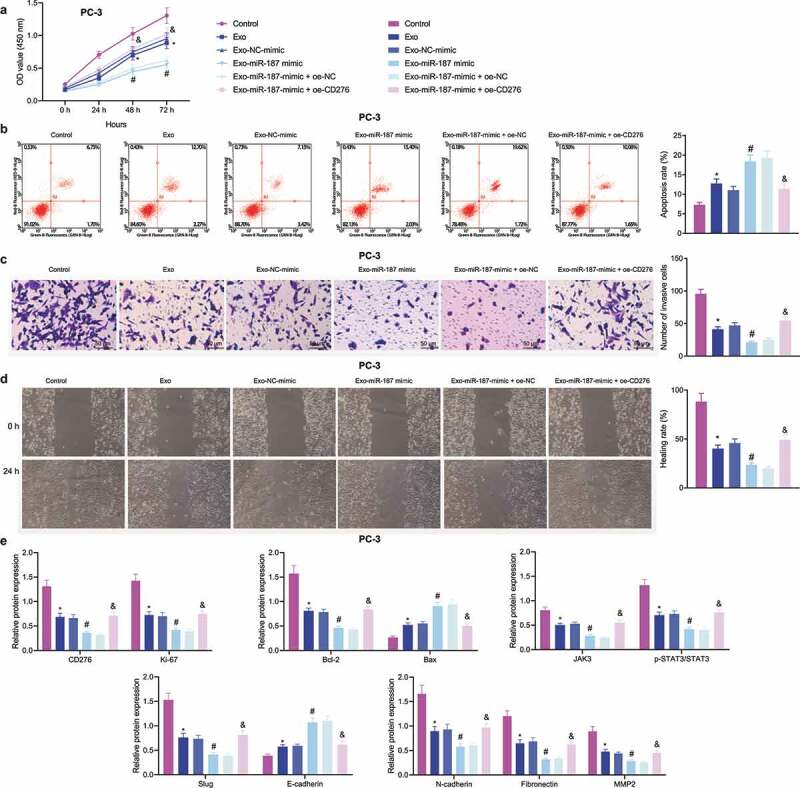
The delivery of miR-187 by hBMSC-exos inhibits the viability, invasion, and migration of prostate cancer cells by inhibiting CD276-dependent JAK3-STAT3-Slug signaling pathway. PC-3 cells were co-cultured with Exo-NC-mimic, Exo-miR-187-mimic or oe-CD276. A, PC-3 cell viability detected using CCK-8. B, PC-3 cell apoptosis detected using flow cytometry. C, PC-3 cell invasion detected using Transwell assay (200×). D, PC-3 cell migration detected using Wound healing test. E, The expression of CD276, JAK3-STAT3-Slug signaling pathway- and apoptosis-related genes in PC-3 cells analyzed by Western blot analysis. The measurement data were expressed as mean ± standard deviation. Cell viability at different time points among multiple groups was compared using two-way ANOVA, and comparison among multiple groups was analyzed using one-way ANOVA with Tukey’s post hoc test. * *p* < .05 *vs*. control PC-3 cells. # *p* < .05 *vs*. PC-3 cells treated with exo-NC-mimic; & *p* < .05 *vs*. PC-3 cells treated with Exo-miR-187-mimic + oe-NC. The experiments were repeated three times.

To verify whether miR-187 could be delivered into PC-3 cells *via* hBMSC-exo to exert its inhibitory effects, the exosomes from hBMSCs overexpressing miR-187 were extracted and co-cultured with PC-3 cells. Proliferative, invasive, and migratory capacities of PC-3 cells co-cultured with hBMSC-exos were weakened, and cell apoptosis was increased. hBMSC-exos overexpressing miR-187 contributed to decreased PC-3 cell proliferative, invasive, and migratory capacities but enhanced PC-3 cell apoptosis. Relative to Exo-miR-187-mimic + oe-NC treatment, the enhanced cell proliferative, invasive, and migratory capacities but limited cell apoptosis were detected in PC-3 cells treated with Exo-miR-187-mimic + oe-CD276 (Figure 6a-d).

Immunoblotting results depicted that the expression of E-cadherin and Bax was elevated while that of CD276, Bcl-2, and JAK3-STAT3-Slug pathway-related genes decreased in PC-3 cells co-cultured with exosomes; these changes were more evident in following Exo-miR-187 mimic. However, Exo-miR-187-mimic + oe-CD276 reversed the effects of Exo-miR-187-mimic (Figure 6e and Supplementary Figure S1F).

Overall, miR-187 delivered by hBMSC-exos targeted CD276 and inhibited JAK3-STAT3-Slug signaling pathway activation, thereby repressing the malignant capacities of prostate cancer cells.

### miR-187 Delivered by hBMSC-exos Suppresses Tumor Growth and Metastasis in vivo by Inhibiting CD276-dependent JAK3-STAT3-Slug Signaling Pathway

**Figure 7.**
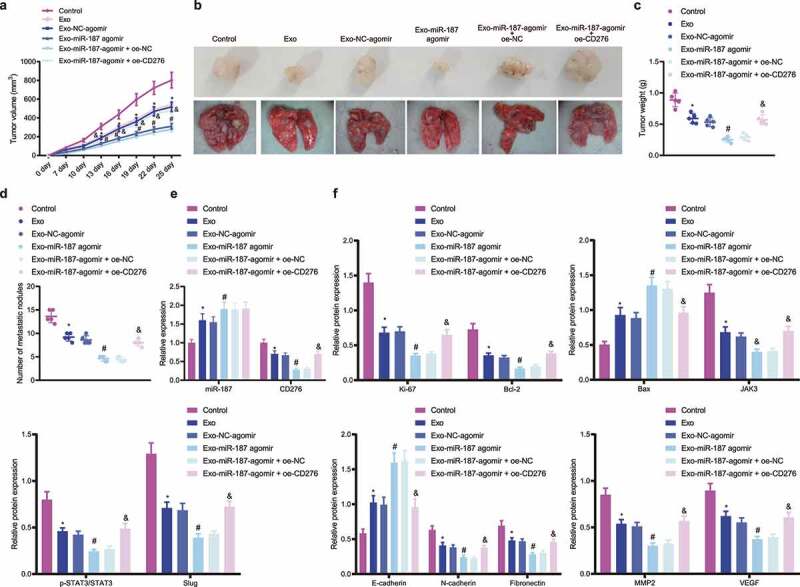
hBMSC-derived exosomal miR-187 inhibits tumorigenesis and metastasis of prostate cancer cells *in vivo* by inhibiting CD276-dependent JAK3-STAT3-Slug signaling pathway. The mice was injected with normal saline, Exo, Exo-NC-agomir, Exo-miR-187 agomir, Exo-miR-187 agomir + oe-NC, or Exo-miR-187 agomir + oe-CD276. A, Tumor volume of mice. B, The representative images of tumorigenesis and lung metastasis of nude mice. C, Tumor weight of mice. D, The number of pulmonary metastatic nodules in mice. E, The expression of miR-187 and CD276 in tumor tissues of mice detected by RT-qPCR. F, Western blot analysis of CD276, Bcl-2, E-cadherin, Bax, and the JAK3-STAT3-Slug signaling pathway-related proteins in tumor tissues of mice. Data at different time points were compared using repeated measures ANOVA and one-way ANOVA was used for the comparison among multiple groups, followed by Turkey’s post hoc test. The measurement data were expressed as mean ± standard deviation. * *p* < .05 *vs*. untreated mice; # *p* < .05 *vs*. mice treated with Exo-NC-agomir; &. *p* < .05 *vs*. mice treated with Exo-miR-187-agomir + oe-NC. n = 5.

The inhibitory role of hBMSC-exo-delivered miR-187 in the growth and metastasis of prostate cancer cells in a PC-3 cell-bearing nude mouse xenograft model was further verified. The results depicted that the volume and weight of tumor as well as the number of pulmonary metastatic nodules were diminished in nude mice treated with Exo or Exo NC-agomir, and a more pronounced reduction was noted following treatment with Exo-miR-187 agomir; however, Exo-miR-187 agomir + oe-CD276 abolished this reduction (Figure 7a-d). Additionally, Exo or Exo-NC-agomir increased the expression of miR-187, E-cadherin and Bax and decreased that of CD276, Bcl-2, and JAK3-STAT3-Slug pathway-related genes in tumor tissues; compared with Exo-NC-agomir, Exo-miR-187 agomir further elevated the expression of miR-187, E-cadherin and Bax but diminished that of CD276, Bcl-2, and JAK3-STAT3-Slug pathway-related genes; Exo-miR-187 agomir + oe-CD276 rescued the effects of Exo-miR-187 agomir (Figure 7e-f and and Supplementary Figure S1G).

Conclusively, miR-187 delivered by hBMSC-exos targeted CD276 and inhibited JAK3-STAT3-Slug signaling pathway, thus retarding the growth and metastasis of prostate cancer cells in nude mice.

## Discussion

BMSCs are adult stem cells with low immunogenicity that are widespread in the bone marrow microenvironment, and recent data have reported that BMSCs can enter the tumor area after intravenous injection.^38,39^ Previous literature has shown that BMSCs may exert their therapeutic activities through secretion of exosomes.^40^ MSC-exos can deliver miR-126 to recipients cells where exogenous overexpression of miR-126 can limit the proliferation of prostate cancer cells in vitro and in vivo; MSC-exos overexpressing miR-126 may work as the therapeutic targeting for prostate cancer.^41^ Based on previous studies, we hypothesized that BMSC-exos could deliver miR-187 to prostate cancer. The current study pinpointed that hBMSC-exos overexpressing miR-187 inhibited prostate cancer through disruption of the targeting CD276-mediated JAK3-STAT3-Slug signaling pathway activation.

It was noted in our work that miR-187 was repressed while CD276 was elevated in prostate cancer. The miRs have been documented to be critical for prostate cancer development *via* oncogenic or anti-oncogenic effects.^42^ More specifically, a decrease of miR-187 expression was measured in the study conducted by Casanova-Salas *et al*.^13^ Also, another study highlighted the diminished miR-187 expression in prostate cancer cells.^43^ CD276 overexpression linked to the poor prognosis of patients with prostatectomy cohorts.^44^ On the other hand, Zang *et al*. also observed the overexpression of CD276 in patients with prostate cancer,^45^ which was similar to our findings. Moreover, the present study demonstrated that miR-187 targeted CD276 and negatively regulated CD276 expression, thus inhibiting the JAK3-STAT3-Slug pathway. Our results were consistent with a recent study which documented that in ccRCC, miR-187 targeted CD276 and decreased the CD276 mRNA expression.^14^ Similarly, the results obtained from a study also proved that upregulation of CD276 resulted in activation of the JAK2-STAT3-Slug pathway in HCC.^19^ Therefore, it can be concluded that the miR-187 inactivates the JAK3-STAT3-Slug pathway by targeting CD276 in prostate cancer.

We also unraveled that hBMSC-derived exosomal miR-187 suppressed the malignant features of prostate cancer cells. Exosomes were released by diverse cells and could be transported between cells by carrying proteins, miRs, lncRNAs, and mRNAs.^8,46^ In addition, exosomes can be derived from hBMSCs and involved in the enhancement of VEGF expression during tumor development.^47^ The exosomal miRs have been highlighted as biomarkers for prostate cancer.^48^ Importantly, the increased enrichment of miR-187-3p is found in MSC-exos,^11^ which was consistent with our results that miR-187 expression was high in the purified hBMSCs-exos. As previously documented, restored expression of miR-187 led to inhibition of cervical cancer cell malignant features.^49^ Another study also clarified the promoting effect of miR-187 on cervical cancer cell apoptosis.^50^ Additionally, the suppression of osteosarcoma cell invasion and migration was observed after miR-187 upregulation in a prior study.^51^

**Figure 8.**
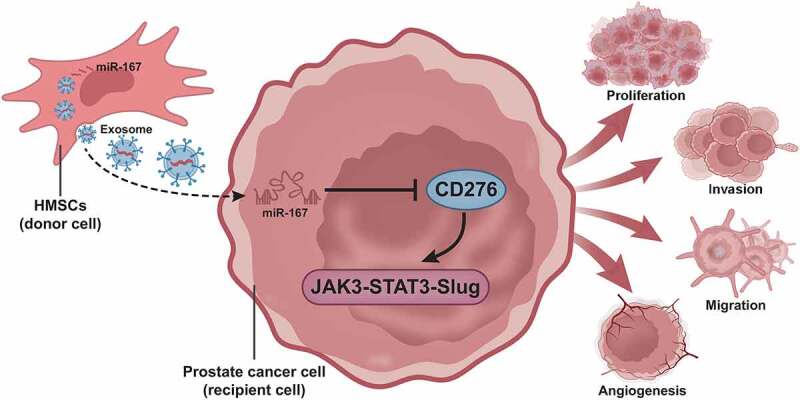
A molecular mechanism map depicting the involvement of hBMSC-derived exosomal miR-187 in the growth and metastasis of prostate cancer. hBMSC-derived exosomal miR-187 targeted CD276 and inactivated the JAK3-STAT3-Slug signaling pathway, thereby suppressing the growth and metastasis of prostate cancer.

To sum up, the present study put the emphasis on the suppressive effect of hBMSC-exos carrying miR-187 on prostate cancer. Our results pinpointed that miR-187 was diminished in prostate cancer tissues and cells and blocked the JAK3-STAT3-Slug signaling pathway by targeting CD276. In addition, we also observed that hBMSC-exos overexpressing miR-187 suppressed cell malignant phenotypes in prostate cancer (Figure 8). Thus, hBMSC-exos overexpressing miR-187 may function as a promising target for the prostate cancer treatment.

## Supplementary Material

Supplemental Material

## Data Availability

The data and materials of the study can be obtained from the corresponding author upon request.
