# Supplementary material for: Suppressive function of bone marrow-derived mesenchymal stem cell-derived exosomal microRNA-187 in prostate cancer
Source: Cancer Biol Ther. 2022 Oct 16;23(1):1–14. doi: 10.1080/15384047.2022.2123675 (PMC9578467; doi:10.1080/15384047.2022.2123675)
Supplement: Supplemental Material [file KCBT_A_2123675_SM5339.zip › KCBT_A_2123675 supplement/Supplementary Table 1.docx]

**Supplementary Table 1** Primer sequences of related genes for RT-qPCR

| Target | Sequence (5'-3') |
| --- | --- |
| miR-187 | F: TCGTGTCTTGTGTTGCAGCCGG |
|  | R: Reverse universal primer |
| CD276 | F: CTCACAGGAAGATGCTGCGT  R: CTCTGGGGTGTGATGGTGAC |
| U6 | F: CTCGCTTCGGCAGCACA |
|  | R: Reverse universal primer |
| GAPDH | F: AATGGGCAGCCGTTAGGAAA |
|  | R: GCGCCCAATACGACCAAATC |

Notes: miR-187, microRNA-187; GAPDH, glyceraldehyde-3-phosphate dehydrogenase; F, forward; R, reverse; RT-qPCR, reverse transcription quantitative polymerase chain reaction.
