# Supplementary figures and images for: Suppressive function of bone marrow-derived mesenchymal stem cell-derived exosomal microRNA-187 in prostate cancer
Source: Cancer Biol Ther. 2022 Oct 16;23(1):1–14. doi: 10.1080/15384047.2022.2123675 (PMC9578467; doi:10.1080/15384047.2022.2123675)

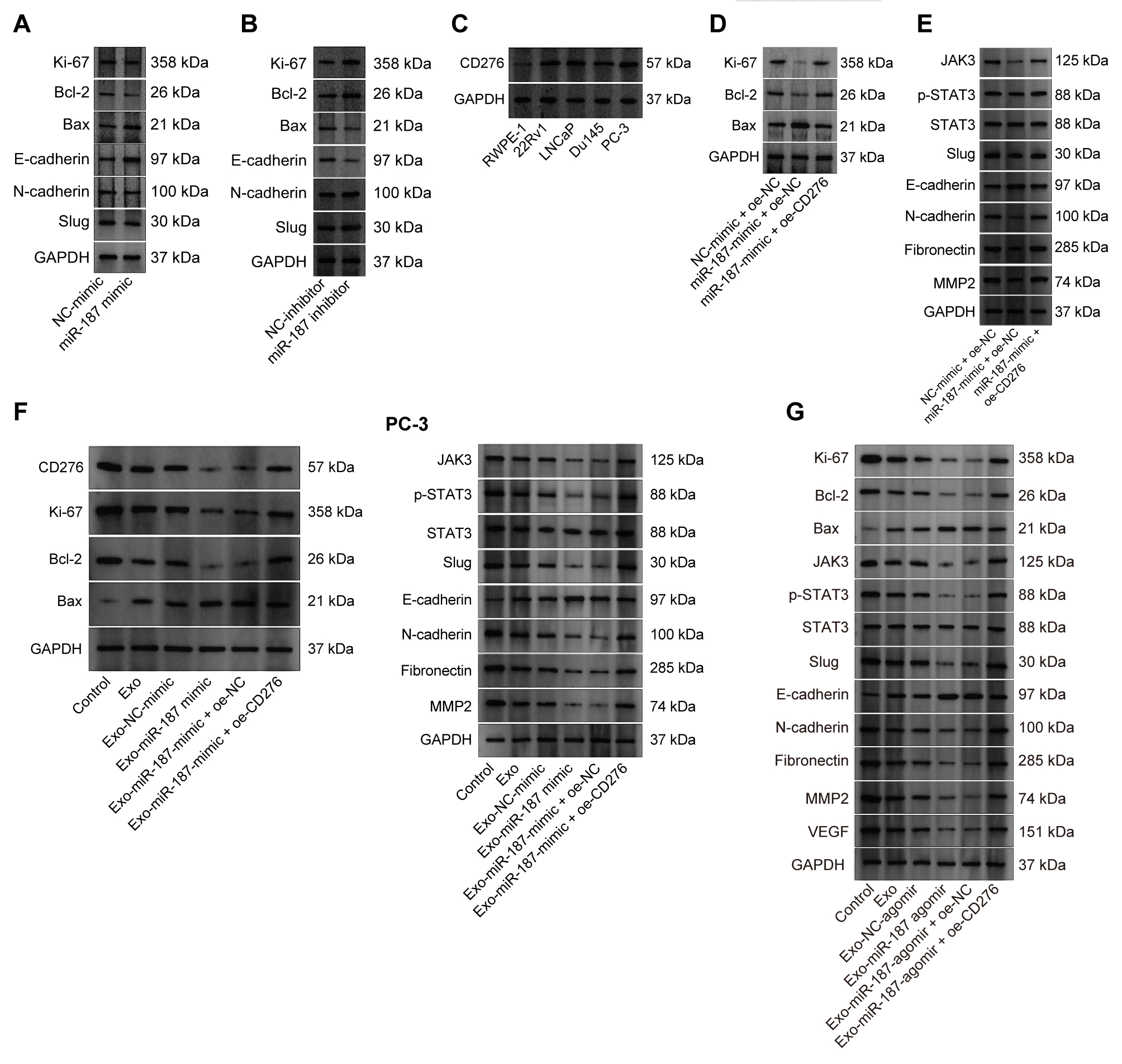

Supplement: Supplemental Material [file KCBT_A_2123675_SM5339.zip › KCBT_A_2123675 supplement/New Figure S1 (1).jpg]
